# Id1 expression in kidney endothelial cells protects against diabetes‐induced microvascular injury

**DOI:** 10.1002/2211-5463.12793

**Published:** 2020-06-26

**Authors:** Shree Sharma, Matthew Plotkin

**Affiliations:** ^1^ Arkana Labs Little Rock AR USA; ^2^ Department of Nephrology John L. McClellan VA Hospital University of Arkansas for Medical Sciences Little Rock AR USA

**Keywords:** diabetes, endothelial cell, Id1, microvascular injury, senescence

## Abstract

The inhibitor of differentiation (Id) transcription regulators, which are induced in response to oxidative stress, promote cell proliferation and inhibit senescence. Inhibitor of differentiation 1 (Id1) expression is limited to endothelial cells (EC) in the normal mouse kidney and is required for a normal response to injury. Endothelial dysfunction leads to the development of diabetic nephropathy, and so, we hypothesized that endothelial Id1 may help protect against hyperglycemia‐induced microvascular injury and nephropathy. Here, we tested this hypothesis by using streptozotocin to induce diabetes in Id1 knockout (KO) mice and WT B6;129 littermates and examining the mice at 3 months. Expression of Id1 was observed to be increased 15‐fold in WT kidney EC, and Id1 KO mice exhibited increased mesangial and myofibroblast proliferation, matrix deposition, and albuminuria compared with WT mice. Electron microscopy demonstrated peritubular capillary EC injury and lumen narrowing, and fluorescence microangiography showed a 45% reduction in capillary perfusion area with no reduction in CD31‐stained areas in Id1 KO mice. Microarray analysis of EC isolated from WT and KO control and diabetic mice demonstrated activation of senescence pathways in KO cells. Kidneys from KO diabetic mice showed increased histological expression of senescence markers. In addition, premature senescence in cultured KO EC was also seen in response to oxidative stress. In conclusion, endothelial Id1 upregulation with hyperglycemia protects against microvascular injury and senescence and subsequent nephropathy.

## Graphical abstract

The inhibitor of differentiation (Id) transcription regulators are induced in response to oxidative stress and inhibit cell senescence. Id1 is expressed in kidney endothelial cells, and levels are increased in response to hyperglycemia. Using streptozotocin‐induced hyperglycemia in Id1 knockout mice, we demonstrate that Id1 protects against microvascular injury and endothelial cell senescence and subsequent nephropathy.

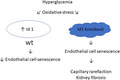

**Abbreviations**
bHLH: basic helix‐loop‐helix
BMP: bone morphogenetic protein
DM: diabetes mellitus
EC: endothelial cells
Id1: inhibitor of differentiation 1
KO: knockout
PAS: periodic acid–Schiff
WT: wild‐type

Microvascular impairment due to endothelial dysfunction is a major complication of diabetes and has been shown to be an early promoter of nephropathy 1, 2. Hyperglycemia adversely affects key endothelial functions including maintenance of vascular tone, permeability, matrix secretion, and angiogenesis. Extensive research has clarified the mechanisms by which this occurs with a central role of reactive oxygen species (ROS) production by NADPH oxidase (Nox) 3 and mitochondrial superoxide production 4, 5 contributing to vascular dysfunction and stimulation of inflammatory and profibrotic molecules, including TGF‐β1.

While hyperglycemia‐induced metabolic derangements and resulting ROS‐mediated endothelial cell (EC) dysfunction have been well documented, it remains unclear how this leads to the development of diabetic nephropathy. In addition to the profibrotic effects of decreased nitric oxide production 6, other proposed mechanisms include endothelial–mesenchymal transition to fibroblasts 7, EC apoptosis 8, or senescence 9, all potentially resulting in microvascular rarefaction and kidney fibrosis.

TGFβ can be counteracted by bone morphogenetic protein (BMP) signaling through a related group of receptors that signal through Smad proteins and downstream induction of Id (inhibitor of differentiation) expression. Ids are a family of 4 (Id1‐4) related proteins with distinct cell‐specific expression and regulation, and function as dominant‐negative regulators of profibrotic basic helix‐loop‐helix (bHLH) transcription factors (reviewed in Ref. 10). Id expression is decreased in response to TGFβ/Smad 2/3 but has a sustained increase in response to BMP/Smad 1/5/8 signaling. Id proteins are induced by oxidative stress 11, 12 and promote EC survival and delay onset of cellular senescence 13, suggesting a potentially important role in preventing EC dysfunction.

The key role of inhibitor of differentiation 1 (Id1) expression in promoting EC survival and preventing capillary loss and fibrosis has been shown in lung 14 and colon 15 injury models. Kidney EC express Id1 at relatively high levels compared with other tissues, but whether or not Id1 has a role in preventing kidney microvascular injury remains unknown. Given the prosurvival effects of increased Id1 expression following oxidative stress, we sought to determine the effects of streptozotocin (stz)‐induced hyperglycemia on endothelial Id1 expression and the effect of Id1 knockout (KO) on EC response to hyperglycemia, and the resulting effect on the development of nephropathy and the mechanisms by which this may occur. We demonstrate that hyperglycemia and oxidative stress increase endothelial Id1 expression. Id1 KO results in endothelial injury and expression of senescence markers in response to hyperglycemia with resulting microvascular damage and nephropathy.

## Results

### Id1 expression is increased in WT EC following streptozotocin‐induced DM and Id1 KO results in DM‐induced EC injury and nephropathy

**Figure 1:**
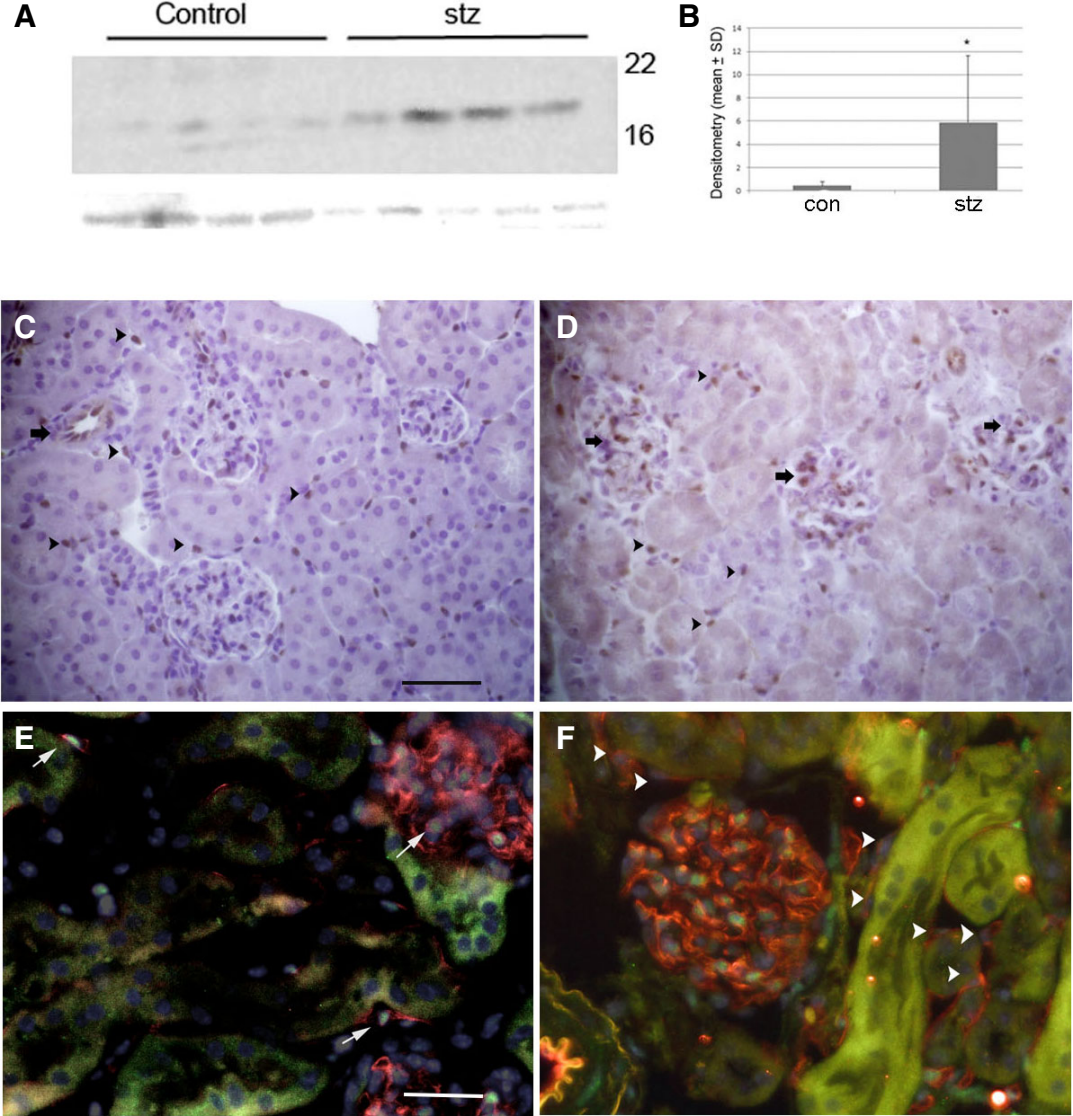
Kidney endothelial Id1 expression is increased following stz‐induced diabetes. (A) Western blot and corresponding densitometry (B) of Id1 levels in whole kidney lysates from 5‐month‐old control (con, *n* = 4) and 3‐month post‐stz‐treated (*n* = 4) WT B6;129 mice. *P < 0.001 (unpaired Student’s *t*‐test). Data presented as mean ± SD. Immunohistochemistry of Id1 expression in WT control (C) and 3‐month post‐stz‐treated (D) kidneys [arrows: arteriolar (C) and glomerular (D) EC, arrowheads: peritubular EC, scale bar = 30 μm C, D]. Immunofluorescence images of Id1 (green) and CD31 (red) labeling (arrowheads: Id1‐negative, arrows: Id1‐positive peritubular EC) in control (scale bar = 50 μm E, F) and stz‐treated (F) mice.

To determine whether Id1 expression changes following stz‐induced diabetes, Id1 was examined by western blot using whole kidney lysates and by immunohistochemistry and double‐labeling immunofluorescence in kidney sections from vehicle‐injected control mice and at 3 months following stz injection. Compared to control 5‐month‐old male B6;129 littermates, Id1 protein levels increased up to 15‐fold following stz‐induced diabetes (Fig. 1A,B). Id1 was detected in peritubular, arteriolar, and glomerular EC in both normal (Fig. 1C) and stz‐treated mice (Fig. 1D), with increased expression in glomerular capillary EC (Fig. 1D,F, EC labeled with CD31) in stz treated compared with control mice (Fig. 1C,E). No Id1 expression was detected in other kidney cell types using these techniques.

**Figure 2:**
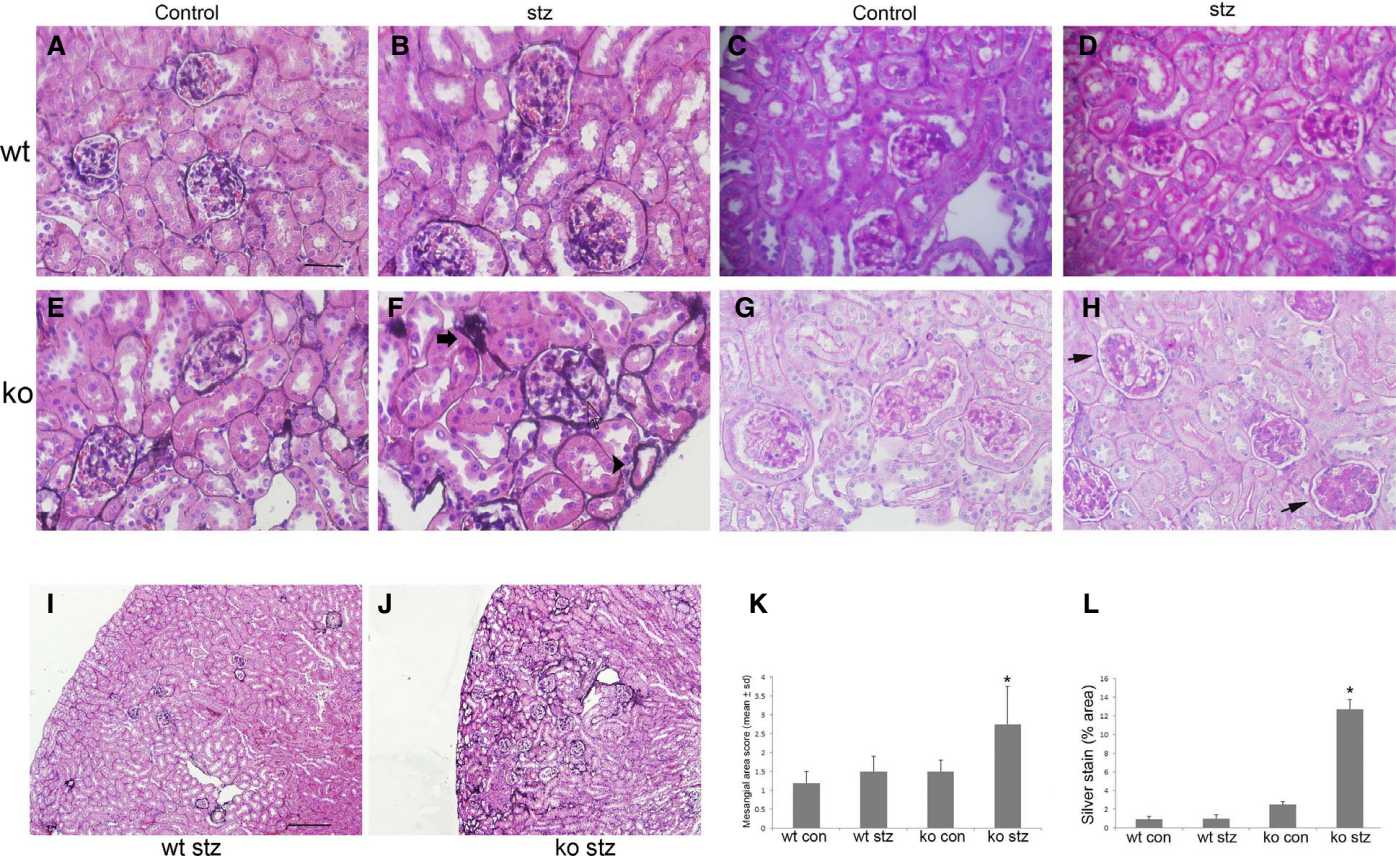
Mesangial cell proliferation and mesangial and peritubular matrix deposition are increased in stz‐treated Id1 KO mice. Methenamine silver stains of kidneys from control (scale bar = 30 μm A–H) and stz‐treated WT (B) and control (E) and stz‐treated (F) Id1 KO mice (arrow: glomerular arteriole, arrowhead: tubular basement membrane, and open arrow: glomerular basement membrane silver staining). PAS stain of kidneys from control (C) and stz‐treated WT (D) and control (G) and stz‐treated Id1 KO (H) mice (arrows: PAS‐positive mesangial matrix accumulation). Original magnification = 400×. (K) Quantification of mesangial cell area (% area 0–25: 1, 25–50%: 2, 50–75%: 3, > 75%, *P < 0.01) and percent area of silver staining (L) score (*n* = 5 mice/group, *P < 0.01, one‐way ANOVA) using imagej software with corresponding low‐power (scale bar = 250 μm I, J) silver‐stained images (I, J). Data presented as mean ± SD.

To determine whether increased Id1 expression has a protective role and prevents hyperglycemia‐induced kidney injury, Id1 KO mice in a diabetic nephropathy‐resistant B6;129 mouse strain were examined and compared with WT mice at 3 months following stz injection (Fig. 2). As previously reported, WT male B6;129 mice displayed mild mesangial matrix expansion by periodic acid–Schiff (PAS) staining (Fig. 2C,D,K) but no other pathological changes by silver staining (Fig. 2A,B,L) 16. In contrast, Id1 KO littermates developed mesangial expansion and mesangial and peritubular matrix deposition by PAS (Fig. 2G,H,K) and silver staining (Fig. 2E,F,I,J,L).

**Figure 3:**
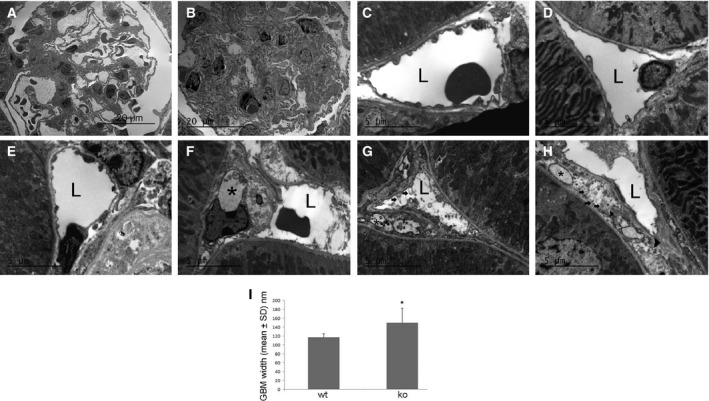
Glomerular basement membrane thickening and endothelial injury occurs in stz‐treated Id1 KO mice. Electron micrographs of sections from stz‐treated WT (A) and Id1 KO (B) glomeruli at low‐power and peritubular capillary endothelial cells from control (C) and diabetic WT (D) and control (E) and diabetic Id1 KO mice (F–H): L = lumen, *vacuoles, G: arrows = membrane ‘blebs’, H: arrows = dissociated ribosomes, arrowhead = swollen endoplasmic reticulum. (I) GBM width measured in 10 capillary loops from two glomeruli/kidney (*n* = 3 kidneys/group), *P < 0.05 (unpaired Student’s *t*‐test). Data presented as mean ± SD. Scale bars = 5 μm (C–H), 20 μm (A, B).

Examination of kidney pathology in diabetic mice by electron microscopy (Fig. 3) also demonstrated increased mesangial matrix deposition in Id1 KO (Fig. 3B) compared with WT glomeruli (Fig. 3A). Peritubular capillary EC demonstrated significant injury in KO mice including cytoplasmic swelling and vacuolization (Fig. 3F‐H), endoplasmic reticulum swelling and disaggregated ribosomes (Fig. 3H), membrane pseudopodia (Fig. 3G), and narrowing of capillary lumens (Fig. 3F‐H) compared to KO control mice (Fig. 3E). EC from WT control (Fig. 3C) and diabetic mice (Fig. 3D) appeared completely normal. Glomerular basement membrane thickness was modestly but significantly increased in KO compared with WT mice (Fig. 3I). These changes occurred despite no difference in average blood glucose levels (WT: 435 ± 75 vs KO: 395 ± 54) over 3 months. These pathological changes resulted in increased proteinuria in control vs stz‐treated mice (albumin/creatinine: 210 ± 110 to 562 ± 580 μg·mg^−1^ vs 144 ± 82 to 188 ± 19 μg·mg^−1^, *n* = 10 mice/group, *P* < 0.05) at 3 months in Id1 KO compared with WT mice.

### Diabetic Id1 KO mice have decreased peritubular capillary perfusion

**Figure 4:**
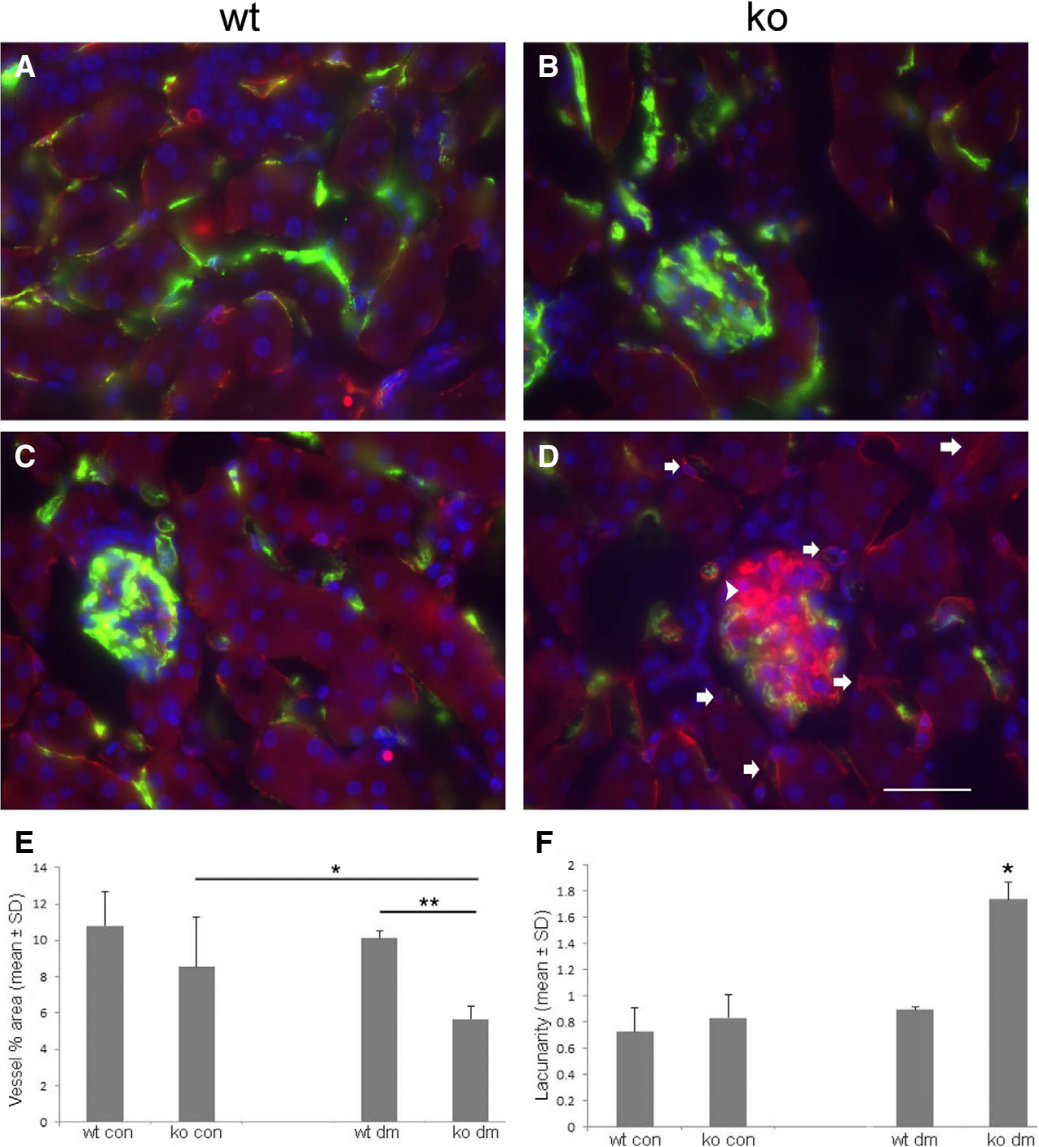
Fluorescence microangiography demonstrates decreased capillary perfusion in Id1 KO kidneys from stz‐treated mice. Representative fluorescence images (green = FluoSphere angiography, red = CD31, blue = dapi) from WT and Id1 KO control (A, B) and diabetic (C, D) mice. (D) Arrows: CD31‐positive, FluoSphere‐negative peritubular capillaries, arrowhead = CD31‐positive, FluoSphere‐negative glomerular capillaries. Vessel area (E: *P < 0.05, **P = 0.0005) and lacunarity (F: *P = 0.0005, ANOVA with Tukey HSD test) quantified by angiotool (NCI) using 10 images/kidney section from five mice/group, scale bar = 30 μm A–D. Data presented as mean ± SD.

The effect of Id1 KO on capillary density following hyperglycemia was determined by fluorescence microangiography 17 to examine perfusion, combined with CD31 staining for capillary density (Fig. 4). Differences in microvasculature perfusion and pattern were quantified for both fluorescent labels using angiotool 18. No significant differences between control WT (Fig. 4A) and control KO (Fig. 4B) and control WT (Fig. 4A) diabetic mice (Fig. 4C) were found. By microangiography, KO diabetic mice (Fig. 4D) had a 45% reduction in microvascular area and a 100% increase in lacunarity, indicating greater pattern variability 19 (Fig. 4E,F). No difference in capillary density was detected by CD31 staining (data not shown). This was associated with a large number of poorly perfused CD31‐positive glomerular and peritubular capillaries (Fig. 4D).

### Gene expression microarrays reveal activation of pro‐inflammatory and fibrotic pathways and genes associated with cell senescence

Whole‐genome microarray analysis was performed to identify molecular pathways responsible for hyperglycemia‐induced damage of the renal vasculature in KO mice. EC from both control and diabetic WT and Id1 KO mice (*n* = 3–4/group) were isolated by intravital fluorescence labeling with a VE‐Cadherin antibody followed by cell sorting. Kidney sections from these mice showed labeling of peritubular and glomerular EC in all four groups (Fig. S1). EC RNA was used for Agilent gene expression microarray analysis. Principal component and hierarchical clustering analysis demonstrated very close correlation of gene expression levels within normal WT and KO EC groups and the presence of one partially dissimilar sample from both the WT and KO diabetic mice (Fig. S2). Analysis of differentially expressed genes revealed a large number of significantly upregulated compared with downregulated genes in EC isolated from KO in comparison with WT control mice (Table S1), consistent with Id1 function as a transcription inhibitor. Id proteins inhibit bHLH transcription factors by both protein dimerization and blocking transcription 20. Multiple HLH factors were significantly increased in KO EC including a compensatory increase in Id4 (Table 1). Pathway analysis using ingenuity® IPA software (Qiagen, Redwood City, CA, USA)  revealed numerous significantly upregulated pathways in KO EC from control mice as expected given the large number of differentially expressed genes. The canonical TGFβ superfamily/Smad signaling pathway was one of the most significantly affected pathways (Fig. S3, *P* = 0.00002), supporting the specificity of the microarray.

**Table 1:** Upregulated HLH factors in Id1 KO EC.

Factor	Fold inc	*P* value
Hand2	7	0.001
E2A	2.6	0.008
E2‐2	3	0.01
HEB	2.4	0.02
TCF 25	2	0.007
TAL1	2.3	0.02
Paraxis	3.7	0.005
HES1	2.6	0.01
HEY 1	3.5	0.01
Id4	3.2	0.003

Additional comparisons focused on differences between WT and KO diabetic mice. Pathway analysis revealed that EC from diabetic KO mice had significant increases in gene expression associated with actin cytoskeleton activation including paxillin, integrin, and adhesion kinase signaling in comparison with diabetic WT mice. Downregulated gene expression included pathways associated with mitochondrial dysfunction, oxidative phosphorylation, and RXR activation, with a common upstream regulation by PPARα, suggesting that Id1 may be a downstream mediator of PPARα signaling (Table 2). In response to the PPARα agonist fenofibrate, MyEnd microvascular EC cultures showed a fivefold increase in Id1 protein levels, further supporting this possibility (Fig. S4). These pathways are identical to those identified by gene expression microarray 21 and proteomics analysis 22 of senescent‐cultured EC. KO diabetic EC had increased inflammatory gene expression including IL‐6 compared with KO control EC. In contrast, no inflammatory genes were increased in WT diabetic EC compared with control EC. Sirtuin 1 expression, a key inhibitor of EC senescent phenotype 23, however, was increased 2.5‐fold in these cells.

**Table 2:** Ingenuity analysis of up‐ and downregulated pathways.

Pathways	*P* value
KO vs WT diabetes upregulated	
Cytoskeleton activation/adhesion	
Paxillin signaling	0.0001
Actin cytoskeleton signaling	0.001
FAK signaling	0.003
PAK signaling	0.003
Integrin signaling	0.005
ILK signaling	0.005
KO vs WT diabetes downregulated	
PPARα signaling	
Mitochondrial dysfunction	0.0001
Oxidative phosphorylation	0.0001
FXR/RXR activation	0.002

Analysis of microarray results comparing KO and WT EC from nondiabetic mice revealed activation of key DNA damage response‐induced senescence pathways in KO cells including p53 and ATM (*P* = 0.0003) and NF‐κB (*P* = 0.0004). Combining gene expression lists from the Reactome 24 and Molecular Signatures Database (Fridman_Senescence_Up) 25 for cell senescence showed upregulation of 59 genes in KO control and diabetic cells including cell cycle regulatory, IGF pathway, MAPK signaling, and cytoskeletal and pro‐inflammatory factor genes that are characteristic of this phenotype (Table S2, *P* = 0.03 using Fisher’s exact test).

### **Increased expression of senescence‐associated **β**‐galactosidase and heterochromatin in Id1 KO diabetic mice**

**Figure 5:**
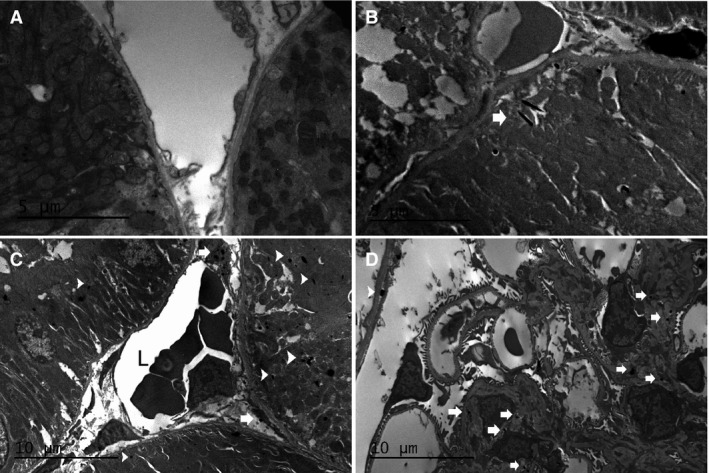
SABG staining is detected in kidneys from diabetic Id1 KO mice. EM images demonstrating X‐gal crystals in tubular epithelial cells (B, arrow, C, arrowheads), endothelial cell (C, arrows), and glomerular mesangial cells (D, arrows). No crystals are detected in WT diabetic kidneys (A). Scale bars = 5 μm (B) and 10 μm (C, D).

Senescence‐associated β‐galactosidase (SABG) expression by the Glb1 gene 26 is one of the hallmarks of cell senescence. Glb1 expression was increased 3.5‐fold (*P* = 0.04) by microarray analysis in KO diabetic EC. To determine whether senescent cells accumulate in Id1 KO diabetic kidneys, tissue was stained for SABG and MacroH2A1.1, a histone enriched in senescence‐associated heterochromatin foci 27 that is used as a marker for senescent cells 28. As shown in Fig. 5, transmission electron microscopy of SABG‐stained kidneys showed that X‐gal crystals were present in epithelial (Fig. 5B), endothelial (Fig. 5C), and mesangial (Fig. 5D) cells in diabetic KO kidneys. No SABG crystals were detected in control or WT diabetic (Fig. 5A) kidneys.

**Figure 6:**
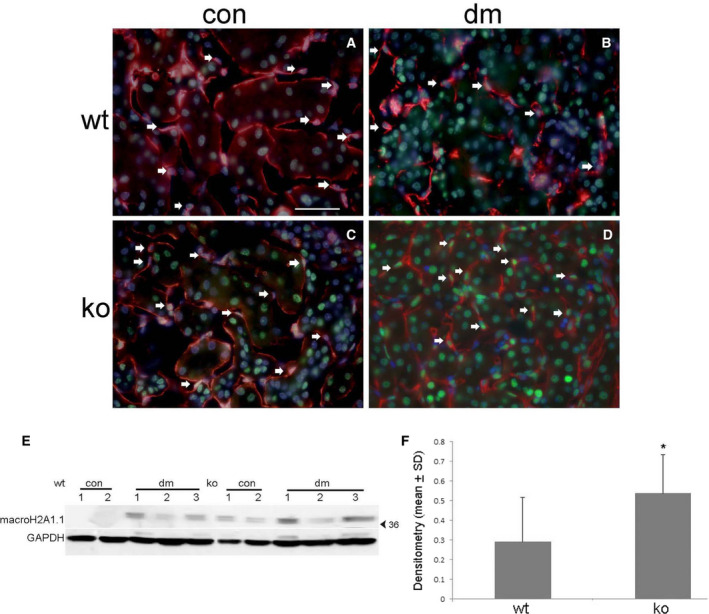
The senescence‐associated heterochromatin marker macroH2A1.1 is increased in diabetic Id1 KO mouse peritubular EC. Immunofluorescence images of macroH2A1.1 (green) and CD31 (red) in WT control (A) and diabetic (B) and Id1 KO control (C) and diabetic (D) kidneys. Arrows = EC nuclei, scale bar = 25 μm A–D. (E) Western blot and corresponding densitometry (F) for macroH2A1.1 expression in control (con) and diabetic (DM) kidneys from WT and id1 KO mice as indicated. *P < 0.05 (unpaired Student’s *t*‐test). Data presented as mean ± SD.

MacroH2A1.1 expression was detected by immunofluorescence (Fig. 6) in tubular epithelial cells in KO controls (Fig. 6C) and both WT (Fig. 6B) and KO diabetic (Fig. 6D) kidneys and was undetectable in WT control cells (Fig. 6A). Peritubular EC expression was also detected in KO diabetic kidneys (Fig. 6D) along with a twofold to threefold increase in expression compared with WT diabetic kidneys by western blot (Fig. 6E,F). No MacroH2A1.1 expression was detected by either technique in WT control kidneys.

### **Increased expression of fibronectin and **α**SMA in Id1 KO diabetic mice**

**Figure 7:**
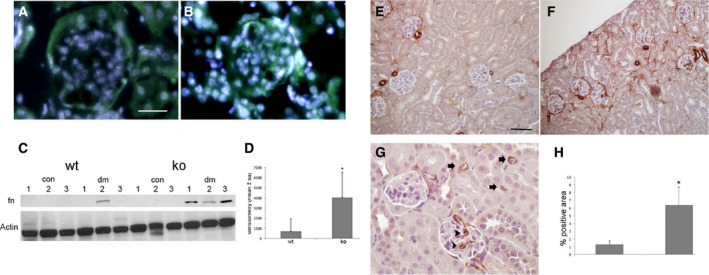
Increased fibronectin and αSMA expression in diabetic Id1 KO kidneys. Immunofluorescence images of fibronectin (green) expression in WT (A) and Id1 KO (B) diabetic kidneys, scale bar = 25 μm A, B. (C) Representative western blot and corresponding densitometry (D) for fibronectin (fn) expression in control (con) and DM kidneys from WT and id1 KO mice (*n* = 5 mice/group) as indicated. *P < 0.05. Immunohistochemical staining for αSMA in WT (E) and Id1 KO diabetic kidneys (F, G, arrows = αSMA‐positive peritubular capillaries, arrowheads = αSMA‐positive glomerular capillaries), scale bar = 50 μm E‐G. (H) Percent area of αSMA‐positive cells (*n* = 5 mice/group, *P < 0.001, one‐way ANOVA) using imagej software. Data presented as mean ± SD.

Expression of the matrix protein fibronectin is another hallmark of senescent cells 29. Increased microarray expression in EC isolated from KO control and diabetic EC was verified by immunofluorescence of kidney tissue (Fig. 7A,B), demonstrating increased glomerular endothelial cell signal in diabetic KO kidneys and by western blot (Fig. 7C,D), demonstrating a fourfold increase in expression in KO diabetic kidneys.

Paracrine effects of endothelial injury were evaluated by examining pericapillary αSMA expression, a marker for pericyte or myofibroblast activation, by immunohistochemistry. αSMA expression was confined to arterioles in WT diabetic kidneys (Fig. 7E). Increased expression (Fig. 7H) was detected in KO diabetic kidneys in both glomeruli (Fig. 7G) and peritubular cells (Fig. 7F), and small number of cortical peritubular capillaries (Fig. 7G). The association between Id1 and αSMA expression was also examined by immunohistochemistry in two genetic models of diabetes including DBA.2Akita and Lep^ob^/WiscJ mice (Fig. S5). DBA.2Akita diabetic mice had nearly undetectable Id1 expression compared with DBA.2 WT and Lep^ob^/WiscJ diabetic mice. Interstitial and glomerular αSMA staining was only detected in DBA.2Akita mice, correlating with results from Id1 KO mice.

### Hyperglycemia‐induced ROS and DNA damage response is increased, and oxidative stress results in cell senescence in Id1 KO EC

**Figure 8:**
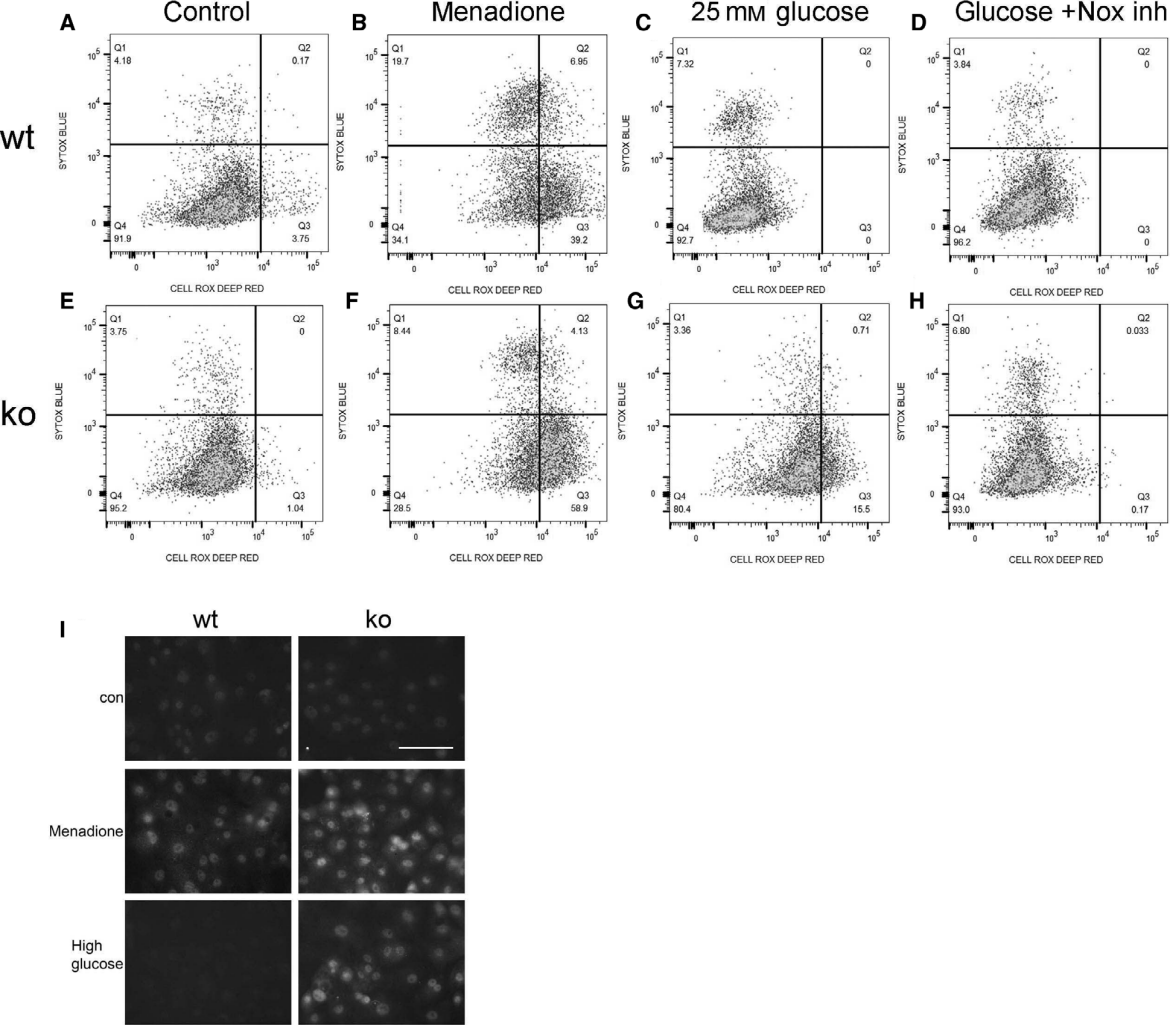
Increased ROS in Id1 KO EC in response to acute hyperglycemia and inhibition with a Nox inhibitor. WT (A‐D) and Id1 KO (E‐H) lung EC treated with 4.5 mm glucose and 20.5 mm mannitol (control), the oxidant menadione (50 μm) as a positive control, 25 mm glucose, or 25 mm glucose + 10 μm Nox inhibitor VAS2870 (Nox inh) 2 h prior to assay with CellROX deep red (*x*‐axis) and SYTOX Blue dead‐cell indicator (*y*‐axis) with percent positive cells shown in each quadrant. (I) Immunofluorescence images for 8‐OHdG expression in WT and KO EC treated as indicated, scale bar = 20 μm all images.

Oxidative stress by mitochondrial and Nox‐induced superoxide production is the key mechanism by which hyperglycemia induces endothelial dysfunction. Microarray analysis demonstrated a 3.7‐fold increase in Nox4 in diabetic compared with control Id1 KO EC, suggesting that Nox‐mediated ROS in Id1 KO EC may contribute to cell dysfunction. Experiments using a ROS fluorescence indicator assay (Fig. 8) demonstrated that acute (2 h) hyperglycemia (25 mm glucose) decreased ROS levels in EC isolated from WT mice (Fig. 8C) compared with control cells (Fig. 8A) but increased ROS in KO cells (Fig. 8G) compared with control KO cells (Fig. 8E), an effect inhibited by pretreatment with a Nox inhibitor (Fig. 8D,H). Positive control cells treated with the oxidant menadione are shown in Fig. 8B,F. No differences in mitochondrial superoxide production were detected using MitoSOX with the same experimental conditions (data not shown). To determine whether hyperglycemia‐induced ROS results in DNA damage, WT and KO cells were stained for 8‐OHdG following treatment with 25 mm glucose for 12 h. Correlating with the ROS assay, the oxidant menadione induced 8‐OHdG in both genotypes, while hyperglycemia‐induced oxidative DNA damage was only detected in KO cells (Fig. 8I).

**Figure 9:**
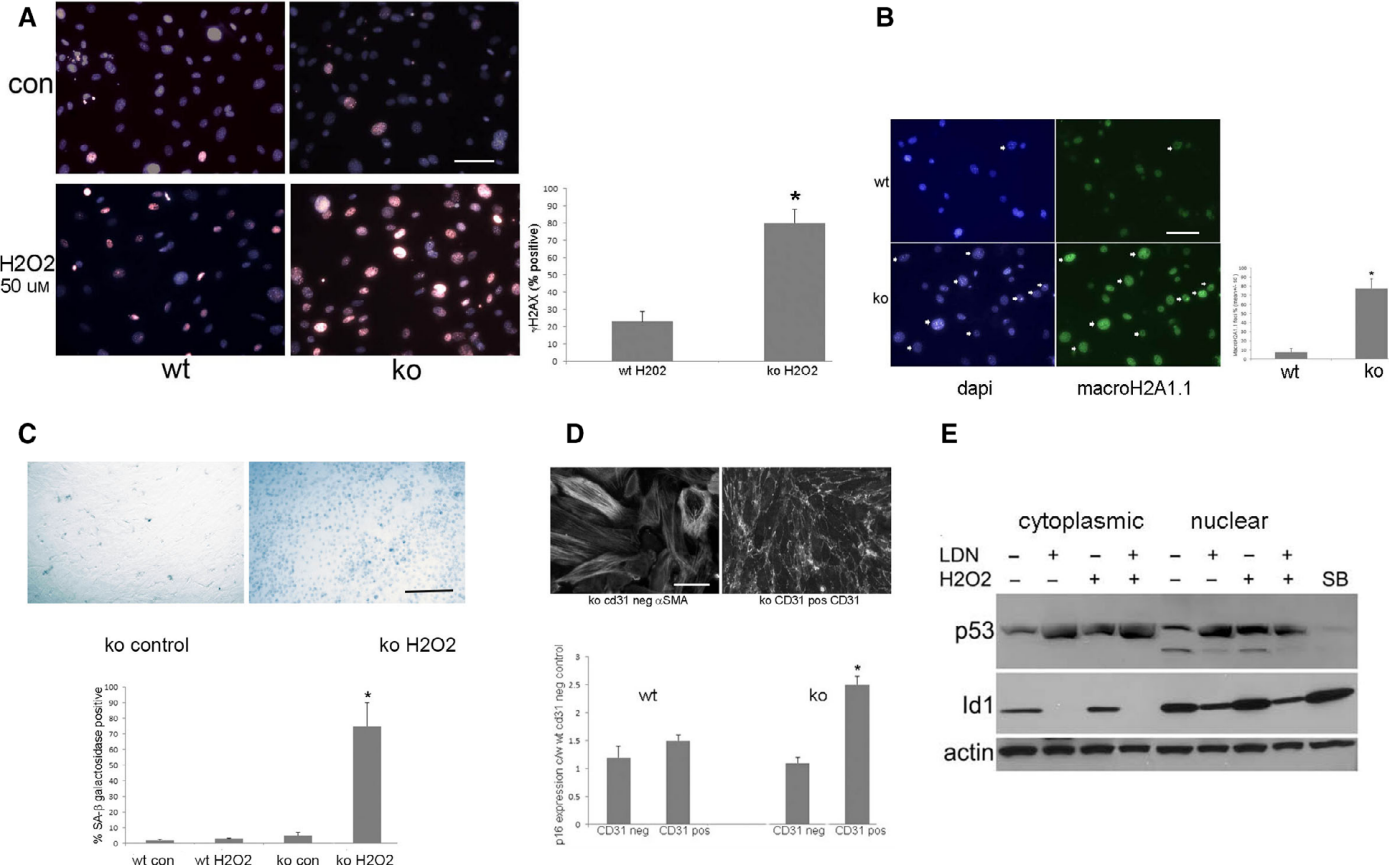
Id1 expression is increased by oxidative stress and Id1 KO results in increased activation of markers of DNA damage, cell senescence, and p53 expression. Cells were treated with 50 μm H_2_O_2_ for 2 h followed by 48‐h incubation in basal medium prior to analysis. (A) Immunofluorescence images of γH2AX expression in lung EC following H_2_O_2_ treatment (*P < 0.01), scale bar = 25 μm all images. (B) Immunofluorescence images of macroH2A.1.1 expression in lung EC 72 h following H_2_O_2_ treatment (*P < 0.01), scale bar = 20 μm. (C) X‐gal staining for SABG expression in control KO and H_2_O_2_‐treated lung EC (*P < 0.001) scale bar = 50 μm all images. (D) Relative p16 expression in WT and KO control (con) and H_2_0_2_‐treated lung CD31‐positive EC and CD31‐negative fibroblasts (*P = 0.04), scale bar = 20 μm all images. (E) Western blot of p53 and Id1 levels in cytoplasmic and nuclear fractions of MyEnd microvascular endothelial cells treated with H_2_O_2_ and Alk antagonists as indicted. (A, B) Results of immunofluorescence, SABG, and p16 qPCR experiments are the mean ± SD of three independent experiments and groups compared using unpaired Student’s *t*‐test.

The effect of Id1 expression on the oxidative stress‐induced DNA damage response and cell senescence was also examined in EC isolated from WT and Id1 KO mice (Fig. 9). H_2_O_2_ (50 μm) treatment resulted in increased γH2AX expression (Fig. 9A), a marker of DNA damage, in KO compared with WT EC. To determine the effect of Id1 KO on oxidative stress‐induced cell senescence, MacroH2A.1.1 (Fig. 9B) and SABG expression (Fig. 9C) was examined in H_2_O_2_‐treated WT and KO cells, demonstrating an eightfold to ninefold and 12‐fold increase, respectively, in KO cells 48 h after treatment. The expression levels of the senescence‐associated cell cycle inhibitor p16^INK4a^ were examined by qPCR, demonstrating a 2.5‐fold increase in expression in H_2_O_2_‐treated KO EC (Fig. 9D). To determine the effect of decreased Id1 levels on oxidative stress‐induced p53 levels, the MyEnd EC line was treated with LDN193189, a BMPr1/Smad 1/5/8 inhibitor followed by treatment with H_2_O_2_. Western analysis (Fig. 9E) demonstrated that decreased Id1 expression resulted in increased p53 expression with no additive effect with H_2_O_2_ treatment. Treatment with SB431542, an Alk5, Smad 2/3 inhibitor, resulted in increased nuclear Id1 level and suppression of p53. Results from these *in vitro* assays correlate with activation of p53‐associated DNA damage response pathways in Id1 KO EC compared with WT EC by microarray analysis (Table S3).

## Discussion

This study demonstrates that Id1 protects against hyperglycemia‐induced endothelial injury and senescence. Endothelial injury in Id1 KO diabetic mice was shown to be associated with mediators of senescence and the senescence‐associated inflammatory phenotype by microarray analysis, and *in vivo* and *in vitro* detection of senescence markers. EC injury resulted in nephropathy by decreased microvascular perfusion and increased matrix deposition.

Changes in vessel architecture in response to hyperglycemia including lumen narrowing and basement membrane thickening have been described in multiple organs including the kidney 30, 31, 32, 33. At a molecular level, cytoskeletal remodeling due to ECM modifications is a key mechanism 34. Our results indicate that Id1 KO leads to significant decreases in capillary perfusion rather that rarefaction due to loss of EC by endothelial–mesenchymal transition or other mechanisms. We initially hypothesized that endothelial Id1 KO would result in EndMT due to unopposed TGFβ and possible sensitization to BMP due to ineffective Smad 1/5/8 signaling as previously demonstrated in Id knockdown epithelial cells 35. Unlike a previous study 7, we detected very few capillaries or interstitial cells (< 1%) that colabeled with CD31 and αSMA, suggesting this was not a mechanism of endothelial injury. Microarray analysis in this and other studies and histological results suggest that the observed perfusion defects may be due to endothelial cytoskeletal activation and changes in matrix including basement membrane thickening and fibronectin secretion. EM analysis demonstrated marked narrowing of peritubular and glomerular capillary lumens associated with enlarged EC cytoplasm that may contribute to the observed hypoperfusion.

Premature senescence in response to hyperglycemia and other forms of oxidative stress has predominately been studied in cell culture. In addition to irreversible cell cycle arrest, senescence is characterized by morphological changes, persistent DNA damage response, and senescence‐associated secretory phenotype, an inflammatory response that is regulated at the transcriptional level by NF‐κB 36, 37. Microarray analysis showed a significant increase in gene expression of the NF‐κB pathway and interferon‐ and interleukin‐regulated genes in Id1 KO EC. Senescence‐associated inflammation contributes to tissue damage and fibrosis in both disease and aging, a mechanism supported by studies showing that deletion of senescent cells in a mouse model of premature aging resulted in reduction of aging‐associated phenotypes 38 and reduced glomerulosclerosis in normal aging 39.

Currently, there is no definitive evidence of *in vivo* EC senescence with kidney aging or injury. Identification of senescent cells, including EC, *in vivo* is technically challenging due to the lack of reliable markers. X‐gal staining for SABG expression has been used to identify senescent EC in atherosclerotic arteries 40 but this technique lacks sensitivity for EC staining in kidney and other tissue sections. Studies have therefore relied upon examining the effects of genetic manipulation of key senescence mediators such as p16INK4a in models of aging and tissue injury 41. Our study uses a combination of previously characterized changes in microarray gene expression, identification of X‐gal crystals using a more sensitive electron microscopy technique 39, and expression of the senescence‐associated heterochromatin marker MacroH2A.1.1. that functions upstream of ATM and is critical for persistent DDR and the inflammatory phenotype during senescence 27.

Id1 downregulation in senescent EC has previously been demonstrated in microarray studies 42. In contrast, induced Id1 expression inhibits senescence 13. Inhibition of cell senescence by Id1 through repression of CDKN2A (p16INK4a) has been demonstrated *in vitro* in numerous cell types including EC 13, 43. ETS2, a transcriptional activator of CDKN2A (p16INK4a), is directly antagonized by Id1 44. Our microarray results showed fourfold to fivefold increases in ETS1 and 2 in KO EC. Although we did not detect increased CDKN2A levels, increases in CDKN2d (p19INK4d), CDKN2Aip, and CDKN1b (p27Kip1) were demonstrated. CDKN2Aip can bind p53 directly and induces cellular senescence through multiple pathways 45, 46 along with these other cell cycle inhibitors 47. *In vitro* studies have also identified mechanisms by which Id1 is downregulated with senescence. Id1 expression is decreased by DEC1, an effector of p53 that is significantly increased with cell senescence 48 and by Smurf2‐induced polyubiquitination 49. In addition to inhibition of p53 and p16INK4a expression, other mechanisms by which Id1 inhibits senescence may exist. EC‐specific KO of SIRT1, an inhibitor of cell senescence, resulted in increased kidney fibrosis in a folic acid injury model 23. Interestingly, microarrays showed that SIRT1 was increased only in WT diabetic EC, suggesting a protective mechanism absent in KO cells. Id1 KO mice used in this study were shown to have increased EC Id4 expression by microarray analysis. The significance of this increased expression is uncertain since Id4 protein expression has only been reported in the CNS and tumor cells 50, 51. A compensatory increase due to Id1 KO, however, cannot be ruled out.

Our microarray results showed a sevenfold larger number of upregulated genes in Id1 KO EC from nondiabetic compared with diabetic mice in comparison with WT cells. Moreover, increased senescence‐associated gene expression compared with WT cells was predominately found in these cells and not Id1 KO EC from diabetic mice. This finding correlates with the absence of MacroH2A1.1 expression in WT control cells and relative lower changes in expression in KO compared with WT diabetic kidneys. In addition to the relative increased expression of these genes in WT EC from diabetic mice, this discrepancy may also be due to the small sample size and increased heterogeneity of the diabetic EC. Although young Id1 KO mouse kidneys are phenotypically normal, increased expression of senescence‐associated genes in these mice may only lead to pathology following cell stress such as hyperglycemia.

Low‐level oxidative stress has been shown to result in ROS generation necessary for propagation of downstream mitogenic and antiapoptotic signals or premature senescence, while higher level stress results in apoptosis. Levels of stress‐induced p53 were mediated by the DNA damage response pathway having a major role in determining which of these final outcomes occur. Previous studies have shown that Id1 and p53 inversely regulate each other, suggesting a mechanism by which Id1 regulates the DNA damage response. To explore this possibility, we used lung EC isolated from both WT and Id1 KO mice and pharmacological treatment of a microvascular EC line to demonstrate that hyperglycemia results in increased ROS in KO cells and that low‐level oxidative stress in KO cells and cells with pharmacological decreases in Id1 expression results in an increased DNA damage response, p53 expression, and markers of cell senescence. Lung EC were used in this study since we and others have found that EC isolated from kidney are very sensitive to the isolation procedure and standard cell culture conditions 52. Isolated cells undergo myofibroblast‐like phenotypic changes within 1–2 days of culture or are ‘contaminated’ with pericytes that are attached to the EC during the purification process. These problems are not seen with isolated lung EC. While differences in gene expression and paracrine growth factor requirements among EC isolated from different organs are noted 53, our experiments focused on response to *in vitro* oxidative stress only. There is no literature to suggest that this response differs among EC isolated from different organs.

In summary, Id1 expression is increased in EC in stz‐induced diabetic mice. Id1 KO results in hyperglycemia‐induced EC injury leading to decreased microvascular perfusion and characteristic diabetic pathology. Microarray analysis suggests that Id1 KO mice develop premature EC senescence and activation of inflammatory pathways, an outcome supported by increased senescence and fibrotic marker expression. *In vitro* experiments demonstrate that Id1 has a protective function against hyperglycemia‐induced ROS and oxidative stress‐induced DNA damage that may be mediated by regulation of p53 levels. EC Id1 expression levels are increased by pharmacological agonists of BMPr1 and PPARα receptors, suggesting that these drugs may be useful in preventing microvascular injury with diabetes through this mechanism.

## Materials and methods

### Mice

Id1−/− mice (B6;129 background) were obtained from R. Benezra and were described previously 15. Mice were genotyped by performing PCR on DNA obtained from tail biopsy, using the primer set for the Id1 locus including 5′ GGTTGCTTTTGAACGTTCTGAACC 3′ (common primer), 5′‐CCTCAGCGACACAAGATGCGATCG3′ wild‐type (WT)‐specific primer, and 5′GCACGAGACTAGTGAGACGTG; 3′ mutant‐specific primer. The PCR results in an 800‐bp and a 500‐bp product for the WT and mutant alleles, respectively.

Diabetes was induced by intraperitoneal stz (Sigma, St. Louis, MO, USA) injection (125 mg·kg^−1^) for two consecutive days as previously described 54. Control mice were injected with equal volumes of 0.1 m Na citrate buffer. Blood glucose was monitored weekly. Mice with glucose levels between 300 and 500 were included in the study. All mice were housed in accordance with guidelines from American Association for Laboratory Animal Care. Research protocols and procedures were evaluated and approved by the CAVHS Institutional Animal Care and Use Committee and followed accepted guidelines for laboratory mouse welfare.

### Western blot

Protein samples were separated by electrophoresis on 5–20% Tris–glycine gels (Novex, Invitrogen/Thermo Fisher, Waltham, MA, USA). Proteins were transferred to nitrocellulose membranes (Bio‐Rad, Hercules, CA, USa) using an XCell miniblot system (Invitrogen). Membranes were blocked with TBST w/5% NFDM for 1 h and incubated overnight at 4° C with primary antibodies. Proteins were detected using HRP‐conjugated anti‐rabbit secondary antibodies at 1 : 2000 dilution (Cell Signaling #7074, Danvers, MA, USA) and SignalFire™ ECL reagent (Cell Signaling). For quantification of protein levels, autoradiographs were scanned, and densitometry was performed with NIH imagej software (Bethesda, MD, USA). Results were corrected for variations in the amount of protein loaded on each lane using corresponding GAPDH or actin levels.

### Immunofluorescence and immunohistochemistry

Immunofluorescence and immunohistochemistry were performed as previously described Ref. 50. Antibodies include the following: rabbit anti‐fibronectin (Novus Bio, NBP1‐91258, Littleton, CO, USA), rabbit monoclonal anti‐Id1 (clone/catalogue number BCH‐1/37‐2; Biocheck, Foster City, CA, USA), rabbit anti‐phospho‐histone γH2AX and macroH2A.1.1 (Cell Signaling Technology #9718 and #12455, respectively), rat anti‐mouse CD31 (clone MEC 13.3, #557355; BD Biosciences, San Jose, CA, USA), and rabbit anti‐αSMA (Abcam, ab 124964, Cambridge, MA, USA). Paraffin sections from DBA.2Akita, DBA.2 WT, and Lepob/WiscJ were provided by DiaComp (NIDDK and University of Washington).

### Fluorescence microangiography

Mice (*n* = 3/group) were anesthetized with isoflurane and perfused with FluoSpheres sulfate 0.02 μm yellow‐green microspheres (Thermo Fisher) conjugated to low melting point agarose (Lonza, Allendale, NJ, USA) exactly as detailed in the protocol by Kramann *et al* 17. Seven‐micrometer cryosections were double‐labeled with rat anti‐mouse CD31 antibody (clone MEC 13.3; BD Biosciences) and detected with Alexa Fluor 647‐conjugated goat anti‐rat IgG (Cell Signaling Technology #4418). Vascular area and lacunarity were measured in separate fluorescence channels for FluoSpheres and CD31 using angiotool software (National Cancer Institute, Bethesda, MD, USA). Fluorescence images were obtained with a Zeiss Axio Imager microscope (White Plains, NY, USA).

### Gene expression microarrays

Mouse kidney EC were isolated by Angiocrine Bioscience (New York, NY, USA). Control and 3‐month post‐stz‐treated diabetic WT mice and Id1 −/− mice (*n* = 3–4/group) were intravitally labeled with VE‐Cadherin Alexa Fluor 647 (Novus Bio, clone BV13). Following cardiac perfusion with M199 media, kidneys were minced and digested with collagenase, dispase, and DNase. Dissociated tissues were filtered, and EC were isolated with a FACSJazz cell sorter. FSC and SSC gates were used to identify the cells, and doublets were excluded, resulting in > 95% pure cells as previously described 53. Cells were sorted directly into TRIzol LE and kept frozen at −80° C until further use.

Microarrays were performed by Phalanx Biotechnology (San Diego, CA, USA). The RNA concentration and purity were checked by OD260/OD280 (≧ 1.8) and OD260/OD230 (≧ 0.7), and the yield and quality were accessed using an Agilent 2100 Bioanalyzer (Agilent Technologies, Santa Clara, CA, USA). Fluorescent aRNA targets were prepared from 0.1 μg total RNA samples using Low Input Quick Amp Labeling Kits (Agilent Technologies). Fluorescent targets were hybridized to Agilent Mouse 8x60K v2 Microarrays (Agilent Technologies). After 16 h of hybridization at 65° C, 10 r.p.m., nonspecific binding targets were washed away with two different washing steps (Wash I 25° C 1 min; Wash II 37° C 1 min), and the slides were scanned using an Agilent G2505C DNA Microarray Scanner (Agilent Technologies). The intensities of each probe were determined using feature extraction 10.7.3.1 software (Molecular Devices, San Jose, CA, USA). The raw intensity of each spot was loaded into r version 2.12.1 to analyze the data. Control probes were first removed before processing the data. Gene clustering, using PCA (principal components analysis) and clustering analysis, was performed on selected gene lists (chosen using the average linkage algorithm) after data transformation and mean centering. The probes with log_2_ ratio ≧ 1 or log_2_ ratio ≦ −1 and *P* value < 0.05 via *t*‐test were defined as differentially expressed genes, and these differentially expressed genes were further used for pathway enrichment analysis.

### Urine creatinine and albumin assays

Urine albumin was measured by ELISA (Exocell, Philadelphia, PA, USA) and normalized to urine creatinine measured by Jaffe reaction (Creatinine Companion, Pointe Scientific, Canton, MI, USA).

### ROS assay

Cells were grown to 70% confluence on 1% gelatin‐coated dishes in low‐glucose (4.5 mm) medium, following treatment with either high glucose (25 mm), menadione (50 μm; Sigma), or high glucose and Nox inhibitor VAS2870 (10 μm; EMD Millipore, Taunton, MA, USA) for 2 h. ROS and cell viability were assayed using a Cell ROX Deep Red Flow Cytometry Kit (Thermo Fisher) and analyzed with a BD LSRFortessa™ flow cytometer.

### Pulmonary endothelial cell isolation

Lungs from 7‐day‐old male pups were digested with 1 mg·mL^−1^ collagenase/dispase (Roche, Indianapolis, IN, USA) in Dulbecco’s modified Eagle’s medium for 30 min at 37° C. Following filtering through 100 μm mesh, cells were plated on 1% gelatin‐coated dishes and grown to 70% confluence. Endothelial cells (EC) were subsequently isolated using magnetic separation with CD31‐coated (clone MEC 13.3; BD Biosciences) followed by CD102 (clone 3C4; BD Biosciences)‐coated goat anti‐rat magnetic beads (New England Biolabs, Ipswich, MA, USA) and cultured in VascuLife EnGS‐Mv endothelial basal medium (Lifeline Cell Technology, Frederick, MD, USA).

### **SA‐**β**‐galactosidase staining**

Cell cultures and tissue slices were fixed for 3 and 25 min, respectively, and stained overnight at 37° C using a commercially available kit (Cell Signaling Technologies). Tissue slices were subsequently postfixed with 4% paraformaldehyde followed by 30% sucrose for cryosections and 4% paraformaldehyde/0.2% glutaraldehyde for EM.

### Quantitative PCR

RNA was isolated from cell cultures using a ReliaPrep Kit (Promega, Madison, WI) with DNAse treatment. cDNA was prepared with a High‐Capacity cDNA Reverse Transcription Kit (Applied Biosystems, Foster City, CA, USA), and comparative qPCR was performed with TaqMan p16 and 18S (endogenous control) primers and a TaqMan Gene Expression Master Mix Assay (Applied Biosystems). Relative quantification (RQ) values were calculated with WT control EC as a reference sample using Applied Biosystems SDS software.

### Statistical analysis

Data were collected from independent experiments and presented as mean ± SD. An unpaired two‐tailed Student’s *t*‐test was used to compare two series of data. One‐way ANOVA for independent samples with a Tukey HSD test for comparison between two groups (www.vassarstats.net/anova1u.html) was used to compare three or more experimental groups. A *P*‐value < 0.05 was considered significant. Microarray pathways were analyzed with the Fisher’s exact test using ingenuity software.

## Conflict of interest

The authors declare no conflict of interest.

## Author contributions

MP designed the project, performed the experiments, analyzed and interpreted the data, and wrote the paper. SS analyzed and interpreted the data.

## Supporting information


**Fig. S1.** EC in WT and ID1 KO control and diabetic kidneys are labeled following intravenous injection of Alexa 647‐conjugated anti‐mouse VE‐cadherin antibody. Confocal fluorescence images of (A) WT control, (B) KO control, (C) WT diabetic and (D) KO diabetic kidneys. Red = VE‐Cadherin, blue = dapi, scale bar = 40 μΔ m.

**Fig. S2.** Principal component (A) and hierarchical clustering (B) analysis of gene expression from microarrays of WT and Id1 KO control (WC and KC) and diabetic (WD and KD) mice.

**Fig. S3.** Ingenuity pathway analysis of TGFβ signaling comparing upregulated gene (purple outline) in KO control vs WT EC. *P* = 0.00002 (Fisher’s Exact Test)

**Fig. S4.** Id1 expression is increased in cultured EC following treatment with fenofibrate. (A) Western blot of Id1 expression in lysates from MyEnd microvascular endothelial cell cultures treated with fenofibrate at indicated concentration (mm) for 3 h.

**Fig. S5.** Decreased Id1 expression in DBA.2Akita diabetic mice correlates with increased αSMA expression. Immunohistochemical staining for Id1 and αSMA as indicated in DBA.2 WT (A, B), DBA.2Akita diabetic (C, D, arrow: arteriole, arrowhead: glomerular capillary, E) and Lepob/WiscJ (F, G) diabetic mice, scale bar = 30 μm.

**Table S1.** Number of significantly up or downregulated EC genes by comparison as indicated. KC = KO control, WC = WT control, WD = WT diabetic, KD = KO diabetic

**Table S2.** Changes in senescence associated gene expression in KO vs WT control EC.

**Table S3.** DNA damage response pathways.
