# Supplementary figures and images for: Id1 expression in kidney endothelial cells protects against diabetes‐induced microvascular injury
Source: FEBS Open Bio. 2020 Jun 26;10(8):1447–62. doi: 10.1002/2211-5463.12793 (PMC7396439; doi:10.1002/2211-5463.12793)

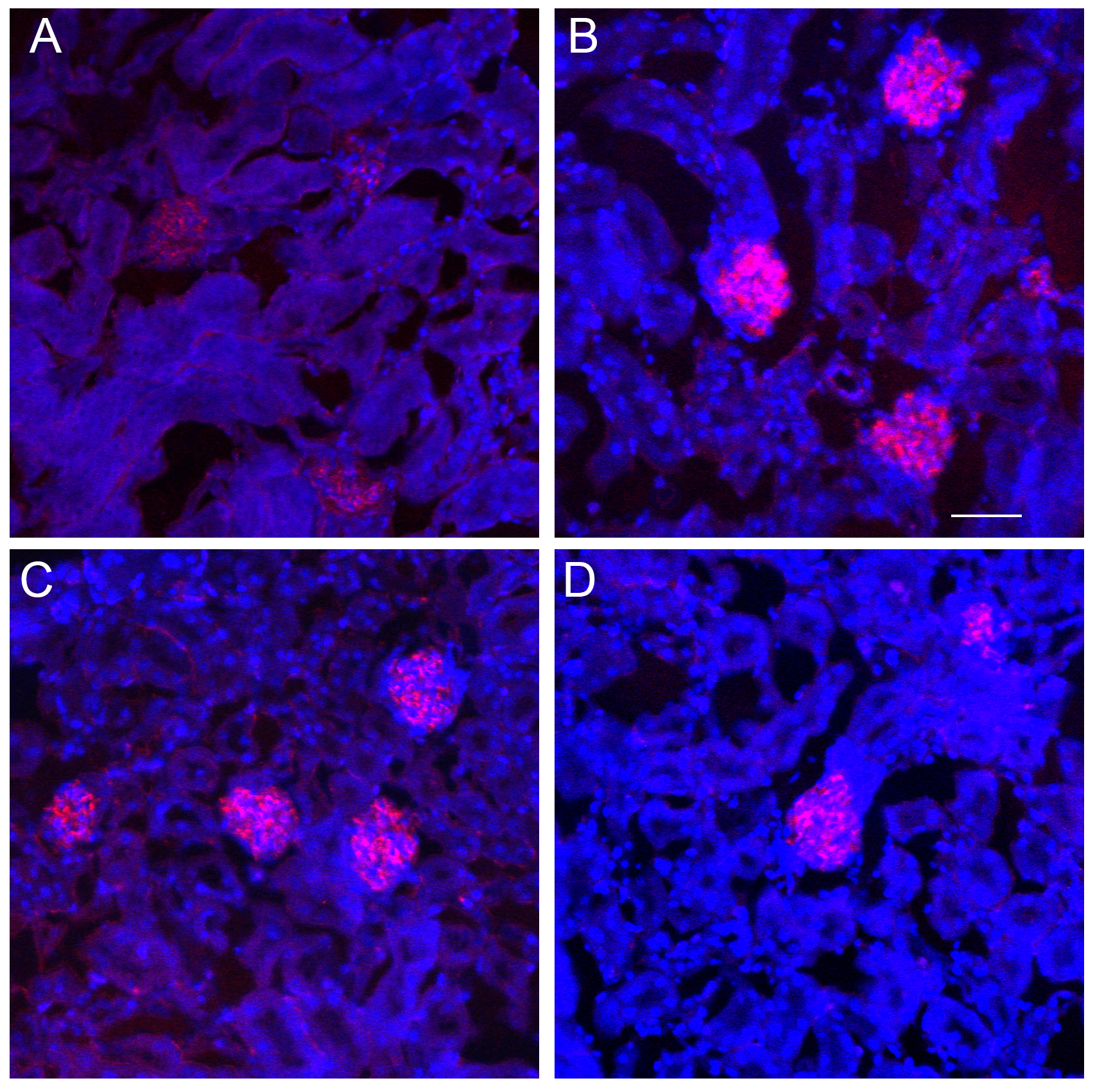

Supplement: Supplementary file 1 — Fig. S1. EC in WT and ID1 KO control and diabetic kidneys are labeled following intravenous injection of Alexa 647‐conjugated anti‐mouse VE‐cadherin antibody. Confocal fluorescence images of (A) WT control, (B) KO control, (C) WT diabetic and (D) KO diabetic kidneys. Red = VE‐Cadherin, blue = dapi, scale bar = 40 μΔ m. [file FEB4-10-1447-s001.tif]

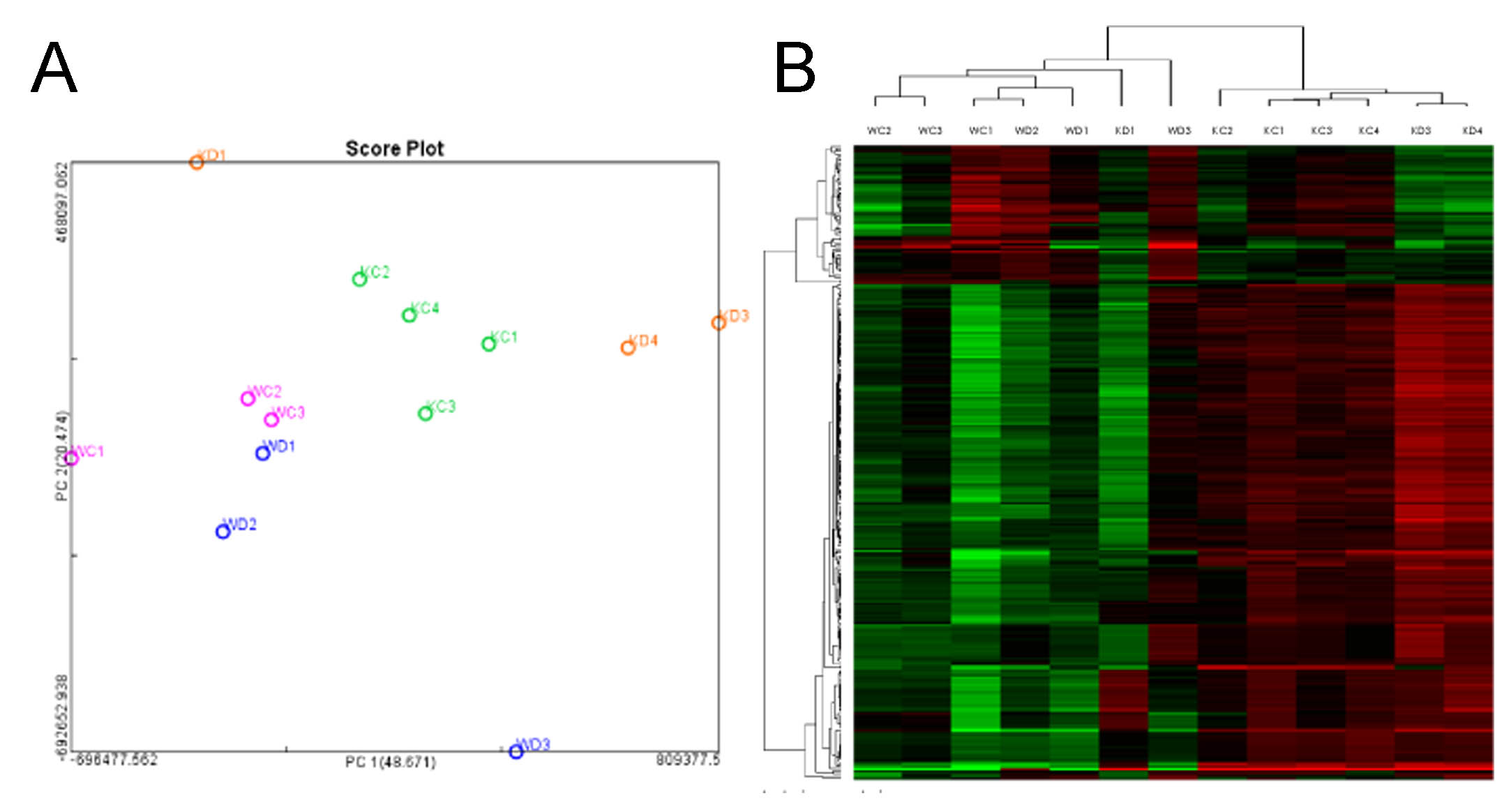

Supplement: Supplementary file 2 — Fig. S2. Principal component (A) and hierarchical clustering (B) analysis of gene expression from microarrays of WT and Id1 KO control (WC and KC) and diabetic (WD and KD) mice. [file FEB4-10-1447-s002.jpg]

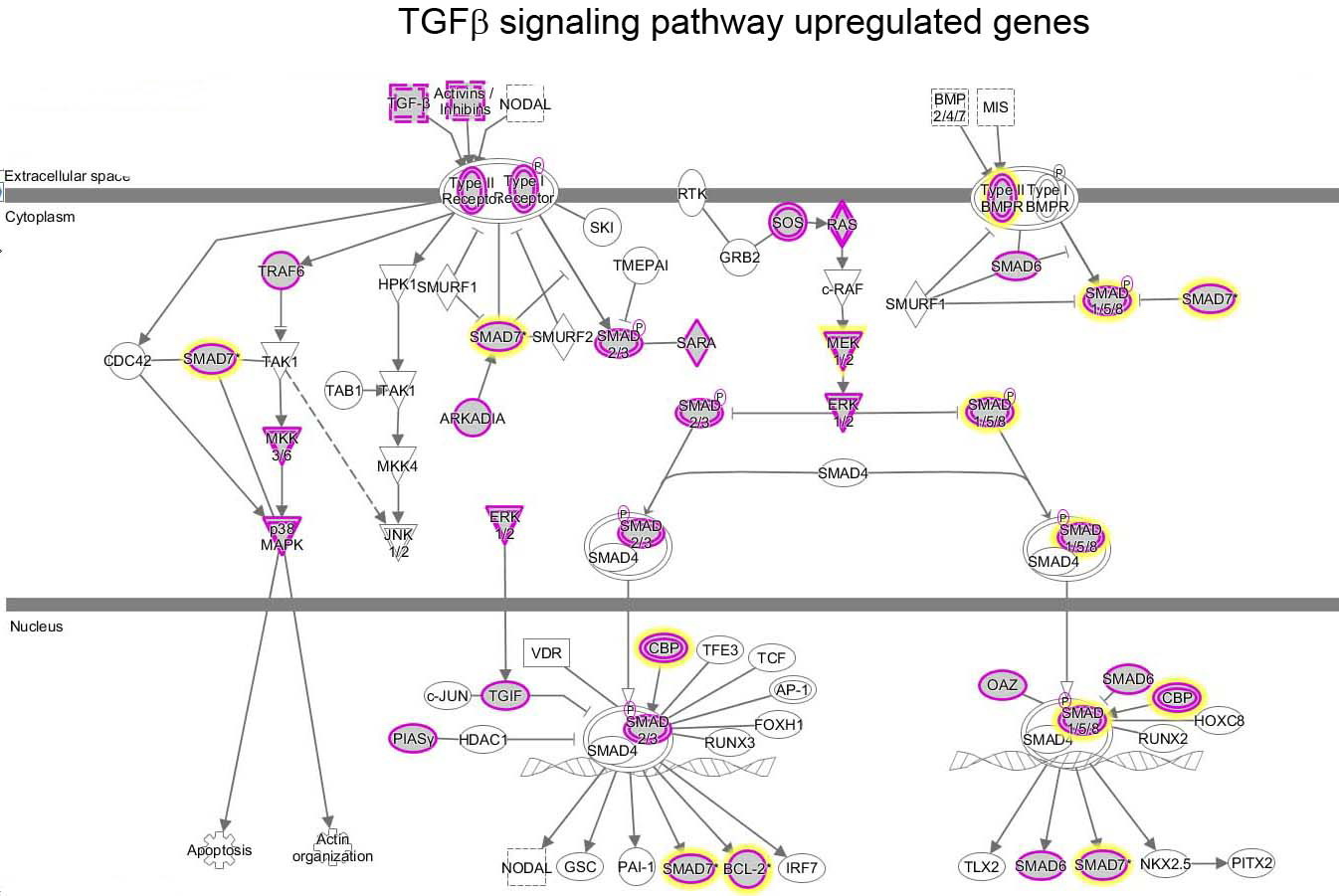

Supplement: Supplementary file 3 — Fig. S3. Ingenuity pathway analysis of TGFβ signaling comparing upregulated gene (purple outline) in KO control vs WT EC. P = 0.00002 (Fisher’s Exact Test) [file FEB4-10-1447-s003.jpg]

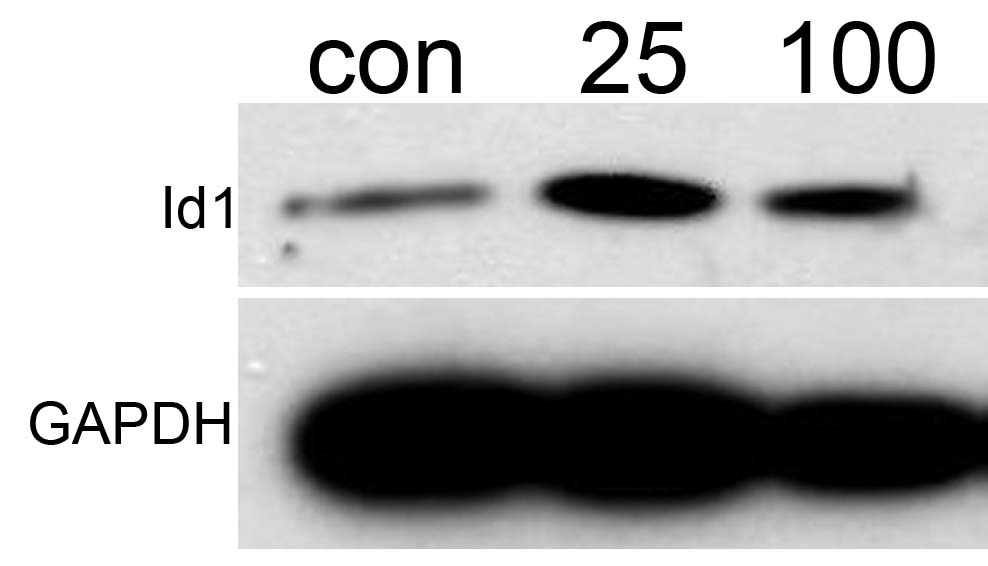

Supplement: Supplementary file 4 — Fig. S4. Id1 expression is increased in cultured EC following treatment with fenofibrate. (A) Western blot of Id1 expression in lysates from MyEnd microvascular endothelial cell cultures treated with fenofibrate at indicated concentration (mm) for 3 h. [file FEB4-10-1447-s004.jpg]

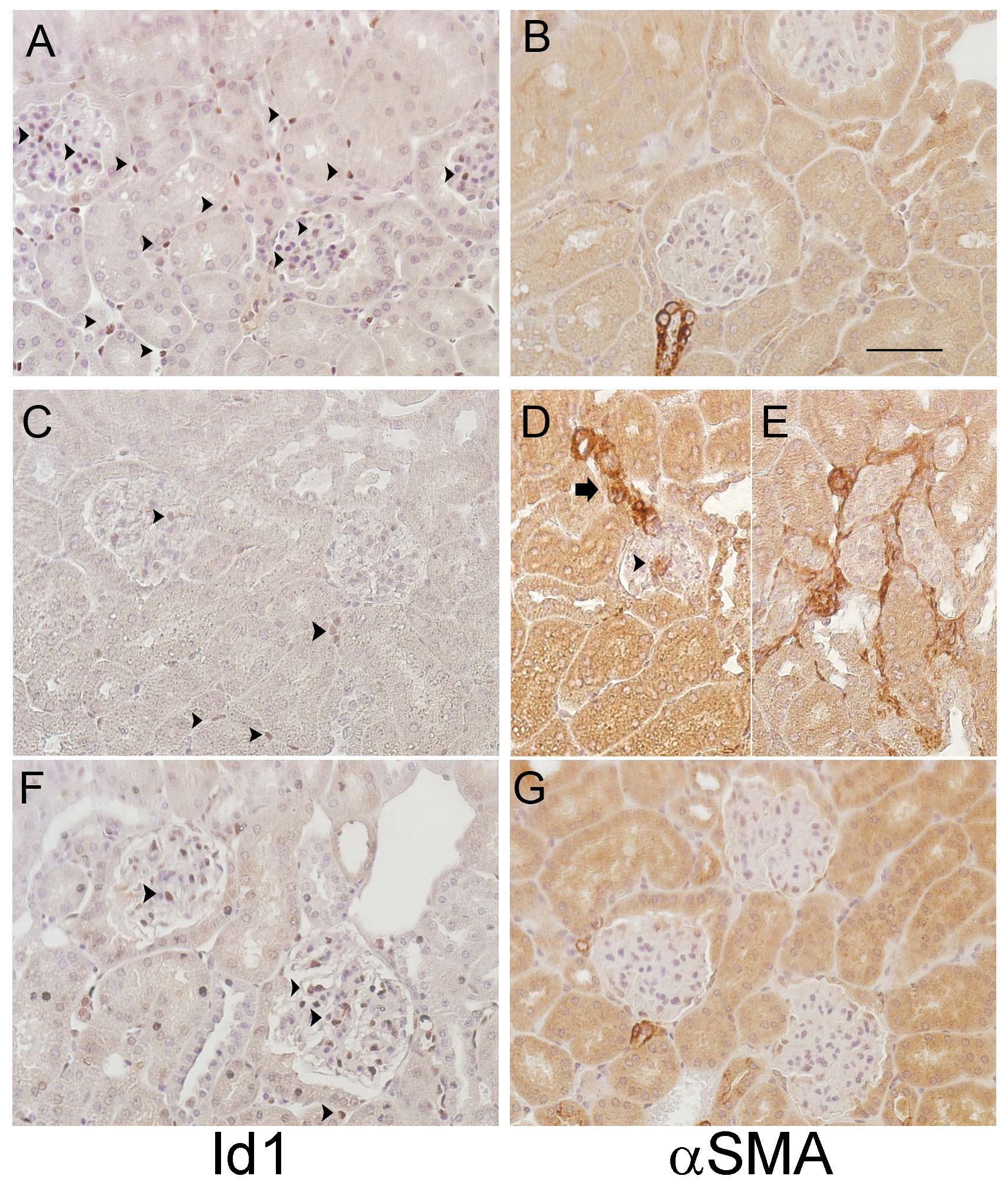

Supplement: Supplementary file 5 — Fig. S5. Decreased Id1 expression in DBA.2Akita diabetic mice correlates with increased αSMA expression. Immunohistochemical staining for Id1 and αSMA as indicated in DBA.2 WT (A, B), DBA.2Akita diabetic (C, D, arrow: arteriole, arrowhead: glomerular capillary, E) and Lepob/WiscJ (F, G) diabetic mice, scale bar = 30 μm. [file FEB4-10-1447-s005.jpg]
