# Supplementary material for: Id1 expression in kidney endothelial cells protects against diabetes‐induced microvascular injury
Source: FEBS Open Bio. 2020 Jun 26;10(8):1447–62. doi: 10.1002/2211-5463.12793 (PMC7396439; doi:10.1002/2211-5463.12793)
Supplement: Supplementary file 6 — Table S1. Number of significantly up or downregulated EC genes by comparison as indicated. KC = KO control, WC = WT control, WD = WT diabetic, KD = KO diabetic [file FEB4-10-1447-s006.docx]

**Supplemental table 1**. Number of significantly up or downregulated EC genes by comparison as indicated. KC= KO control, WC = WT control, WD = WT diabetic, KD = KO diabetic

| Comparison | Upregulated | Downregulated |
| --- | --- | --- |
| KC/WC | 7633 | 201 |
| WD/WC | 493 | 97 |
| KD/KC | 398 | 182 |
| KD/WD | 1468 | 463 |
