# Supplementary material for: Id1 expression in kidney endothelial cells protects against diabetes‐induced microvascular injury
Source: FEBS Open Bio. 2020 Jun 26;10(8):1447–62. doi: 10.1002/2211-5463.12793 (PMC7396439; doi:10.1002/2211-5463.12793)
Supplement: Supplementary file 7 — Table S2. Changes in senescence associated gene expression in KO vs WT control EC. [file FEB4-10-1447-s007.docx]

**Supplemental table 2**: Changes in senescence associated gene expression in KO vs WT control EC

|  | Fold inc | p-value |
| --- | --- | --- |
|  |  |  |
| Molecular Signatures Database | | |
|  |  |  |
| Bcl2 | 14.02194 | 0.036166 |
| Igfbp5 | 11.48037 | 0.000732 |
| Esm1 | 9.333658 | 0.031095 |
| Igfbp3 | 7.489447 | 0.048742 |
| Rab5b | 7.455424 | 0.015043 |
| Cited1 | 6.971109 | 0.005538 |
| Eif2ak1 | 6.178709 | 0.010144 |
| Tnfaip2 | 5.998212 | 0.002103 |
| Tspyl5 | 5.093982 | 0.031209 |
| Eif2ak4 | 4.628462 | 0.009494 |
| Cxcl12 | 4.547109 | 0.020674 |
| Aldh1a3 | 4.512909 | 0.031126 |
| Tnfaip3 | 3.92431 | 0.012222 |
| Filip1 | 3.78468 | 0.044171 |
| Tgfb1i1 | 3.658673 | 0.026492 |
| Vim | 3.477078 | 0.047144 |
| Eif2ak3 | 3.34168 | 0.001263 |
| Cdkn2aip | 3.28192 | 0.035354 |
| Ciz1 | 3.27652 | 0.00896 |
| Rgl2 | 3.180105 | 0.010396 |
| Map2k3 | 3.122574 | 0.000325 |
| Cdkn2d | 3.054884 | 0.008948 |
| Ctgf | 2.951734 | 0.044567 |
| Hbs1l | 2.940188 | 0.001027 |
| Sparc | 2.930155 | 0.040886 |
| Ing1 | 2.864105 | 0.029949 |
| Rac1 | 2.79023 | 0.003399 |
| Rb1 | 2.765712 | 0.00591 |
|  |  |  |
| Reactome Database |  | |
|  |  |  |
| Cdk6 | 9.600548 | 0.017561 |
| Rps6ka2 | 8.030578 | 0.011789 |
| Mapk10 | 6.627318 | 0.034111 |
| Pot1a | 6.486277 | 0.031805 |
| Cdc26 | 5.833572 | 0.010348 |
| Phc3 | 5.482816 | 0.018961 |
| Ep400 | 5.069988 | 0.002499 |
| Ets2 | 5.063139 | 0.004454 |
| Rint1 | 4.985233 | 0.01368 |
| Hist1h3a | 4.524982 | 0.007201 |
| Phc2 | 4.421709 | 0.033542 |
| Cbx6 | 4.323994 | 0.00439 |
| Ets1 | 4.229176 | 0.008168 |
| Terf2ip | 4.109477 | 0.001586 |
| Mapk1 | 3.958946 | 0.001056 |
| Cabin1 | 3.878615 | 0.012119 |
| Scmh1 | 3.777986 | 0.031365 |
| Nfkb1 | 3.773374 | 0.00292 |
| Anapc5 | 3.562227 | 0.049002 |
| Cdc23 | 3.536452 | 0.036507 |
| Kdm6b | 3.446138 | 0.003511 |
| Lmnb1 | 3.415221 | 0.005359 |
| Anapc1 | 3.399854 | 0.008783 |
| Cdkn1b | 3.308226 | 0.001557 |
| Hira | 3.207994 | 0.038982 |
| Map2k3 | 3.122574 | 0.000325 |
| Suz12 | 2.980695 | 0.043534 |
| Terf2 | 2.91751 | 0.008242 |
| Anapc15 | 2.880342 | 0.012197 |
| Map3k5 | 2.875484 | 0.008736 |
| Mapkapk5 | 2.875273 | 0.001034 |
| Pot1b | 2.780939 | 0.042459 |
| Rb1 | 2.765712 | 0.00591 |
