# Supplementary material for: Id1 expression in kidney endothelial cells protects against diabetes‐induced microvascular injury
Source: FEBS Open Bio. 2020 Jun 26;10(8):1447–62. doi: 10.1002/2211-5463.12793 (PMC7396439; doi:10.1002/2211-5463.12793)
Supplement: Supplementary file 8 — Table S3. DNA damage response pathways. [file FEB4-10-1447-s008.docx]

Supplemental table 3: DNA damage response pathways

| KC v WC upregulated pathways | | |
| --- | --- | --- |
|  |  |  |
|  |  | p-value |
| ATM Signaling | | 0.0003 |
| p53 Signaling | | 0.0003 |
| NF-κB Signaling |  | 0.0004 |
