# Supplementary material for: Id1 expression in kidney endothelial cells protects against diabetes‐induced microvascular injury
Source: FEBS Open Bio. 2020 Jun 26;10(8):1447–62. doi: 10.1002/2211-5463.12793 (PMC7396439; doi:10.1002/2211-5463.12793)
Supplement: Supplementary file 9 [file FEB4-10-1447-s009.docx]

**Supplemental figure legends**

**Figure S1** EC in WT and ID1 KO control and diabetic kidneys are labeled following intravenous injection of Alexa 647-conjugated anti-mouse VE-cadherin antibody. Confocal fluorescence images of (A) WT control, (B) KO control, (C) WT diabetic and (D) KO diabetic kidneys. Red = VE-Cadherin, blue = dapi, scale bar = 40 μm.

**Figure S2** Principal component (A) and hierarchical clustering (B) analysis of gene expression from microarrays of WT and Id1 KO control (WC and KC) and diabetic (WD and KD) mice.

**Figure S3** Ingenuity pathway analysis of TGFβ signaling comparing upregulated gene (purple outline) in KO control vs WT EC. p = .00002 (Fisher’s Exact Test)

**Figure S4** Id1 expression is increased in cultured EC following treatment with fenofibrate. (A) Western blot of Id1 expression in lysates from MyEnd microvascular endothelial cell cultures treated with fenofibrate at indicated concentration (mM) for 3h.

**Figure S5** Decreased Id1 expression in DBA.2Akita diabetic mice correlates with increased αSMA expression. Immunohistochemical staining for Id1 and αSMA as indicated in DBA.2 WT (A, B), DBA.2Akita diabetic (C, D, arrow: arteriole, arrowhead: glomerular capillary, E) and Lepob/WiscJ (F, G) diabetic mice, scale bar = 30 μm.
